# Cognitive Neuroscience in Alpine Skiing: Introducing Computational Sports Medicine for Performance Optimization

**DOI:** 10.1111/sms.70188

**Published:** 2026-01-11

**Authors:** Carl‐Johan Boraxbekk, Matej Supej, Hans‐Christer Holmberg

**Affiliations:** ^1^ Department of Neurology, Institute of Sports Medicine Copenhagen (ISMC) Copenhagen University Hospital Bispebjerg Copenhagen Denmark; ^2^ Institute for Clinical Medicine, Faculty of Medical and Health Sciences University of Copenhagen Copenhagen Denmark; ^3^ Faculty of Sport University of Ljubljana Ljubljana Slovenia; ^4^ Division of Machine Elements Luleå University of Technology Luleå Sweden; ^5^ Department of Physiology and Pharmacology, Biomedicum C5 Karolinska Institutet Stockholm Sweden

**Keywords:** athlete development, brain, predictive modeling, working memory updating

## Abstract

While sport psychology has long emphasized mental and cognitive aspects of performance, sports medicine has traditionally focused on musculoskeletal and physiological aspects, largely overlooking the brain's central role in athletic performance. This narrative review aims to bridge this gap by introducing Computational Sports Medicine, a novel framework that integrates cognitive neuroscience with established physiological and biomechanical measures. Using alpine skiing as a primary example, this review examines the critical role of working memory updating in dynamic environments, discusses how neural processes enable adaptation, and proposes Computational Sports Medicine as a unifying predictive framework. This approach moves beyond descriptive analysis to provide objective, quantifiable metrics, testable models, and the ability to simulate “what‐if” scenarios for proactive intervention. Practical implications for training include developing sport‐specific cognitive tasks, individualizing variability in motor and cognitive learning, and leveraging technologies like virtual reality and wearable sensors. The review primarily targets elite and sub‐elite athletes, for whom cognitive and environmental demands are most pronounced. This brain‐inclusive framework offers a personalized approach to performance optimization, injury prevention, and safe return‐to‐play decisions, positioning the brain as the central organ to the future of sports medicine.

## 1 Introduction

Despite the brain's central role in every aspect of athletic performance, from rapid decision‐making under time pressure to the precise activation and coordination of motor actions, sports medicine has traditionally focused on the body below the neck. Consensus statements, training guidelines, and return‐to‐play protocols prioritize musculoskeletal health, cardiovascular conditioning, and peripheral injury prevention, leaving the cognitive and neural processes that enable elite performance underrepresented [1]. While sport psychology has long emphasized mental and cognitive aspects of performance, these insights have rarely been translated into a brain‐focused framework for training, injury prevention, and rehabilitation within sports medicine. In the present paper, we use alpine skiing as an example of elite athletic performance in a dynamic and high‐stakes environment, characterized by rapid perceptual and motor demands, strong external forces, and substantial injury risk. This sport demands an extraordinary integration of physical and cognitive skills, requiring skiers to execute precise motor sequences at high speed while simultaneously processing complex, rapidly shifting environmental cues [2, 3]. For decades, alpine skiing performance analysis primarily emphasized biomechanics and physiology [4, 5]. In contrast, cognitive neuroscience research has revealed the critical role of neural processes in elite sports [6], providing insights into how the brain processes information, makes decisions, and controls movement under pressure. Throughout this paper, we use cognitive abilities to denote general mental capacities such as attention and working memory, and cognitive functions to refer to specific operational components within these abilities, such as updating or inhibition. In some areas of sports medicine, a cognitive neuroscience perspective is increasingly being recognized, for example to understand and manage anterior cruciate ligament (ACL) injuries [7]. Traditionally viewed as a biomechanical injury, ACL injuries are now understood to involve neurocognitive components, encompassing pre‐injury factors and post‐rehabilitation effects at the neural level [8, 9, 10, 11]. Despite an increased focus on developing dual‐task tests that include motor and cognitive performance aspects [12], sports medicine largely remains a “shoulder‐downwards” discipline. In this context, our review aims to highlight the need for a brain‐inclusive framework capable of integrating cognitive, neural, and physiological perspectives into one coherent performance model, to fully capture the complexity of elite performance.

The extreme demands of alpine skiing [4] offer a powerful illustration of the critical role cognitive neuroscience plays in understanding and optimizing athletic performance. The sport's inherent variability, from fluctuating snow conditions and visibility to constantly changing course layouts, requires rapid and continuous adaptation [3, 13, 14]. We will focus on the concept of updating, which is the capacity to monitor and revise working memory representations in response to new information [15]. This cognitive function allows athletes to maintain accurate and flexible internal models of the environment, discard outdated information, and adjust motor programs in real time, directly impacting their ability to navigate complex courses effectively [16]. Although many other cognitive functions are crucial in sports and rarely operate in isolation [15], updating is particularly central to the dynamic demands of alpine skiing performance.

The aim of this narrative review is to address the striking disconnect between brain and body in sports medicine research, despite their inseparable physiological unity, which includes, both neural and somatic processes. First, we examine the role of working memory updating in the high‐speed, complex environment of alpine skiing. Second, we introduce Computational Sports Medicine as a novel framework to integrate cognitive neuroscience with traditional physiological and biomechanical measures. Third, we discuss the practical applications, challenges, and future directions of this brain‐inclusive approach to optimizing both athletic performance and safety.

## 2 Narrative Review Process

Given the novelty of Computational Sports Medicine, a narrative review was chosen to synthesize and conceptually integrate findings across cognitive neuroscience, biomechanics, and sports medicine. The process followed recommended practices for narrative reviews [17]. A search of PubMed and Web of Science was performed using combinations of the keywords: “working memory,” “updating,” “neural mechanisms,” “computational modeling,” “alpine skiing,” and “elite sport.” In line with this conceptual aim, studies were then selected for relevance rather than exhaustive inclusion. Findings were thematically organized into subheadings reflecting core topics identified during synthesis.

## 3 Working Memory Updating and Related Cognitive Functions

Executive functions (EFs) are higher‐order cognitive processes essential for goal‐directed behavior, problem‐solving, and adaptation to novel situations. Miyake et al. [15] proposed a three‐factor model of EFs, identifying updating, inhibition, and shifting as distinct yet interrelated components. Updating, the main focus in this review, refers to the continuous monitoring and rapid revision of information held in working memory. It involves replacing outdated or irrelevant information with new, relevant data to maintain an accurate internal representation of the environment or task [18]. This dynamic process is critical in sports requiring constant attention and flexible responses. For example, in alpine skiing, an athlete must quickly adjust motor programs in response to unfolding events. One such event might be an unanticipated lateral skid during a carved turn caused by abrupt contact with the ice surface.

## 4 The Brain in Updating

Theoretical advancements in understanding cognitive functions are increasingly complemented by computational models which provide mechanistic accounts of how information is maintained, manipulated, and updated within neural networks (e.g., [19]). Brain imaging studies, particularly using functional magnetic resonance imaging (fMRI) and electroencephalography (EEG), have identified a distributed neural network supporting working memory updating. Key brain regions include the dorsolateral prefrontal cortex (DLPFC), the posterior parietal cortex, basal ganglia, and cerebellum, structures that jointly maintain, select, and update task‐relevant representations [20].

The DLPFC plays a central role in maintaining and manipulating information in working memory, as well as in exerting top‐down control over information selection [20]. These functions are critical for athletes, allowing them to attend to task‐relevant cues while suppressing irrelevant stimuli, such as focusing on skiing performance rather than the presence and activities of spectators during Olympic Games competitions. The posterior parietal cortex contributes to the spatial and attentional components of working memory [20], supporting spatial awareness and navigation in complex sports environments. In alpine skiing, these functions enable adaptation to dynamic course demands such as changes in slope inclination, variable turn radii, jumps or altered gate setups [21, 22]. The striatum, particularly through its interactions within the basal ganglia, is hypothesized to play a role in “gating” information into and out of working memory, controlling which information gets updated [23]. This gating mechanism prevents irrelevant information from overloading working memory, ensuring rapid access to task‐relevant information. What appears to be effortlessly performed by elite skiers when constantly managing race challenges, such as changes in snow conditions, ruts, ridges, or sudden shifts in visibility (sun, shade, flat light), is thought to be governed by an intricate and precisely orchestrated machinery at the neural level.

Beyond working memory, repeated exposure to specific stimuli, like vibrotactile input, leads to updated and more precise representations in sensory cortices, enhancing perceptual discrimination [24]. This highlights a fundamental mechanism by which the brain continuously updates its internal models of the environment, a capability critical for skill acquisition and adaptation in sports. In alpine skiing, such updating enables athletes to anticipate and respond to terrain irregularities, subtle changes in snow texture, and/or visual conditions.

Importantly, studies on working memory training indicate that updating mechanisms can be enhanced through practice. Adaptive training protocols have shown training‐induced neural plasticity within frontal, parietal and striatal brain regions, indicating that the brain's updating mechanisms can be enhanced through 5 weeks of practice [18, 25]. A primary challenge in training in general is the difficulty of achieving transfer to untrained skills, and updating training is no exception [26]. Transfer most likely occurs when the trained and transfer tasks rely on overlapping neural networks [18, 27]. This principle should also guide work on transferring improved updating skills into sport. Thibault et al. [28], for example, demonstrated transfer from tool learning to language skills due to shared syntaxes and neural processes. Interestingly, when training produces robust effects, substantial long‐term benefits can also be observed on transfer tasks [25, 29, 30].

To overcome the well‐known limitations of training‐induced transfer, elite skiers adhere to the principle of specificity, designing practice environments that closely mimic competitive conditions to maximize the transfer from practice to performance. A similar approach should guide cognitive training, by developing sport‐specific tasks that target both motor and cognitive demands. Developing such tasks represents a crucial future avenue, requiring collaboration between practitioners and cognitive neuroscientists, as general cognitive tests often demonstrate limited value in predicting elite sports performance [31, 32]. Harnessing decades of research within motor and cognitive training and applying it with a focus on neural mechanisms and sport specificity, may open the door to interventions that enhance performance in elite athletes.

### 4.1 Updating in Alpine Skiing

Alpine skiing presents a dynamic and cognitively demanding environment where the ability to rapidly and accurately update internal representations is paramount [14]. Athletes operate at high speeds, navigating complex courses with constantly changing external conditions [4, 21], requiring continuous cognitive and motor adaptation. Updating is critical across multiple performance phases, from pre‐race planning to real‐time execution. It occurs at two timescales: short‐term updating of working memory enables immediate, moment‐to‐moment adjustments, and longer‐term updating of internal models, reflects learning and adaptation over time.

During course inspection, skiers engage in an active cognitive exercise, slowly progressing down the course while encoding the gate sequence (e.g., rhythm, variations, and delayed gates) and mapping terrain features, including various pitch transitions—such as breakovers (changes from flat to steep inclination) and rolls—and variable snow‐surface conditions [14, 21]. Although the emphasis differs across disciplines, slalom and giant slalom focus more on rhythm and line, whereas super‐G and downhill prioritize terrain reading and speed management, the core inspection process remains fundamentally similar and culminates into a tactical plan built upon a continuously updated three‐dimensional mental model of the course. As athletes advance, preliminary hypotheses about gate rhythm, slope gradient, and the optimal line are iteratively refined through incoming visual, proprioceptive, and auditory cues, prompting micro‐adjustments to the intended trajectory and body position. For example, a segment that initially appears to permit a direct line may conceal a subtle roll or soft patch of snow requiring line, stance and timing adjustments. This process exemplifies short‐term updating, shaped by prior expectations and informed by long‐term knowledge gained through years of training [33]. Between runs, the course is reset, and if conditions change significantly (e.g., from the sun melting the snow), both re‐initialization and rapid model updating are required, demanding cognitive flexibility. Once the race begins, these models must be reconfigured in real time as the environment diverges from inspection. Skiers execute highly practiced motor sequences while processing dynamic sensory input, requiring rapid, moment‐to‐moment updating under extreme time pressure. Several key scenarios illustrate this:

#### 4.1.1 Snow Conditions

The interaction between ski and snow is highly variable, driven by shifts in snow microstructure, temperature, and moisture content, as well as speed and load [34, 35]. Encountering an unexpected icy patch requires immediate motor program update to maintain speed and balance.

#### 4.1.2 Visibility

Fog, flat light, or shadows reduce perceptual clarity, making it difficult to discern terrain features or the precise location of gates [14]. Skiers must update spatial awareness, shifting reliance toward proprioceptive feedback and pre‐race models, and integrating this into the ongoing action.

#### 4.1.3 Course Deterioration

Later starting skiers often face a deteriorated and less predictable course [36]. Consequently, they must update their internal model in real time, adjusting their line and technique for these challenging conditions.

Effective performance relies on the continuous integration of sensory feedback (visual, proprioceptive, vestibular) with motor control [37]. Efficient updating keeps internal models aligned with external reality, enabling adaptive adjustments that maintain speed and control. Failures in updating may manifest as delayed reactions, technical errors, missed gates, time loss, and increased injury risk. Beyond cognitive aspects, updating efficiency is modulated by physiological and psychological states such as fatigue, arousal, and stress. These internal bodily signals influence mental processes and behavior, directly impacting the capacity for adaptive adjustments. For example, physiological arousal indexed by heart rate influences updating efficiency in an inverted‐U shaped manner. While moderate arousal facilitates attentional focus and cognitive flexibility, excessive sympathetic activation can impair prefrontal control and inhibit information processing [38]. Thus, updating capacity represents a critical point where brain, body, and environment converge to determine performance and safety.

## 5 Updating of Motor Programs

Beyond the cognitive representation of the course, updating is fundamental to the adaptive execution of motor programs. While elite skiers rely on highly practiced, almost automatic motor sequences, their execution is far from rigid. Instead, it is characterized by continuous, subtle adjustments driven by sensory feedback. In computational terms, this aligns with predictive coding frameworks, in which the brain constantly generates predictions about incoming sensory information based on internal models of the body and environment, built upon prior experience and expectations [39, 40].

When actual sensory feedback deviates from these predictions, a “prediction error” is generated. This error signal is then used to update the internal models, refining both the sensory predictions and the motor commands that generated them [37]. For an alpine skier, this means that every micro‐deviation from the expected trajectory or sensation (e.g., a slight slip, an unexpected bump, a change in ski pressure, a subtle shift in balance) generates a prediction error. These errors are immediately used to update the ongoing motor commands, leading to constant, subtle adjustments in body position, pressure distribution, and edge angle. This continuous loop of prediction, error detection, and model updating allows for highly adaptive and precise motor control, enabling skiers to maintain balance and optimal line even in the face of unexpected perturbations.

Interestingly, prediction‐error updating does not occur in isolation; it is constrained by the course's biomechanics. From a computational perspective, the brain's internal models aim to minimize trajectory error while limiting the mechanical energy cost of corrective actions. On steep sections, where substantial potential energy is available between gates [41, 42], skiers can implement larger, more abrupt corrections, at expense of kinetic energy, because the vertical drop enables rapid re‐acceleration that offsets temporary speed losses [22]. In contrast, on flat terrain or during transitions, where mechanical energy is limited, prediction errors must be corrected more economically; the motor system prioritizes gradual, low‐cost adjustments to conserve velocity, since lost speed (kinetic energy) cannot be readily regained. Ultimately, prediction‐error updating in alpine skiers likely reflects a continuous, state‐dependent, multi‐objective optimization that balances sensory precision with mechanical‐energy efficiency, dynamically tuned to local course demands.

For coaches and practitioners, this perspective highlights why technical drills should be terrain specific. For example, athletes may need to train more aggressive, high‐cost corrections on steep slopes while emphasizing smooth, economical adjustments on flats. By framing these differences through prediction‐error updating and energetic constraints, training can be better aligned with the brain's natural optimization strategies.

## 6 Computational Sports Medicine: A Novel Framework

Despite major advances in physiology and biomechanics, current approaches in sports medicine often lack the formal tools to capture the complex, dynamic interplay between brain, body, and environment. Research has traditionally been correlational, group‐based, and largely reactive, focusing on observable outcomes rather than underlying mechanisms. This “shoulders‐downward” perspective has yielded invaluable insights into musculoskeletal and cardiovascular performance, but it struggles to explain or predict how athletes adapt under uncertainty, why individuals respond differently to identical training loads, or how the brain plays a role in performance but also sport injuries and return‐to‐play protocols.

**FIGURE 1:**
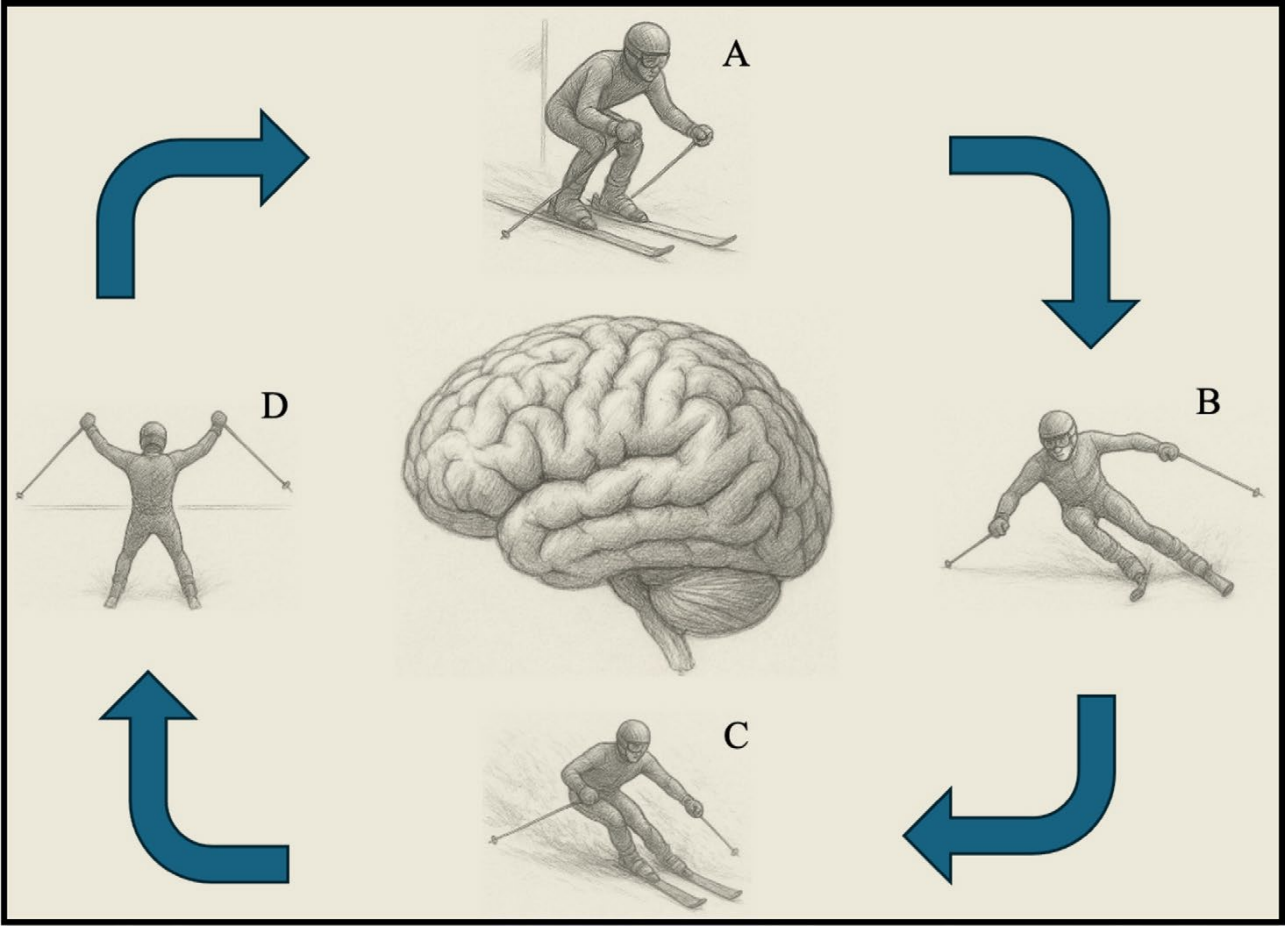
The brain as the central organ in alpine skiing performance: a computational sports medicine perspective. This figure illustrates the continuous, dynamic role of the brain in an alpine skier's performance, from start to finish. The central brain highlights its integrative function, constantly processing information and updating internal models. Each skier represents a critical phase where cognitive and motor processes, as understood through Computational Sports Medicine, are paramount for adaptation and optimal performance. A: Pre‐race optimization: At the start gate, the brain activates highly practiced motor programs and the tactical plan formed during course inspection. It prepares for rapid updating based on initial sensory input, balancing speed and control. B: Mid‐course correction: An unexpected perturbation (e.g., an icy patch) generates a prediction error. The brain rapidly updates internal models and motor commands, making immediate, subtle adjustments to maintain balance and line. C: Cognitive adaptation under pressure: Sudden changes in visibility (e.g., flat light) demand rapid updating of spatial awareness. The brain shifts reliance to proprioceptive feedback and pre‐race models, managing stress to prevent cognitive overload and maintain decision accuracy. D: Fatigue management and sustained performance: Approaching the finish line, as physiological and mental fatigue accumulate, the brain works to maintain optimal updating efficiency and motor control. It prioritizes critical adjustments to prevent errors and ensure a strong finish, balancing energy conservation with precision.

Other fields have faced similar challenges. Computational neuroscience and psychiatry have transformed their disciplines by moving from descriptive, symptom‐based categories to formal, testable models of brain function and dysfunction [43, 44]. These approaches treat the brain as a predictive machine that constantly updates internal models of the body and environment [45]. In sport science, related efforts include complex systems perspectives on injury [46] and calls for artificial intelligence integration into sports medicine [47]. Yet no unifying framework has brought these strands together. See Figure 1 for an illustration of the brain as the central organ in Alpine skiing performance.

We propose Computational Sports Medicine as an integrative framework that unifies cognitive, neural, biomechanical, physiological, psychological, and environmental data through computational modeling. Its goal is to generate formal, testable accounts of performance and injury risk by quantifying how specific factors (e.g., fatigue, stress, snow conditions) affect decision thresholds, updating rates, or motor variability. Importantly, these models are falsifiable because they yield quantitative predictions (e.g., that higher updating efficiency predicts reduced motor variability under sensory perturbation), which can then be empirically tested. Such testability bridges theory and applied practice, allowing researchers and clinicians to validate or refine models through real‐world data. They also allow for simulation of “what‐if” scenarios, enabling proactive intervention rather than reactive response. Importantly, this framework accommodates individual differences, offering tailored predictions rather than “one‐size‐fits‐all” guidelines. In short, it moves sports medicine into a brain–body–environment era, where athlete development is guided not only by observation but by formal models of how performance emerges and adapts under real‐world conditions.

Alpine skiing illustrates the potential of this approach. Modern monitoring already generates vast heterogeneous datasets including Global Navigation Satellite Systems tracking, motion capture, force plates, wearable sensors, sleep and wellness metrics, cognitive performance tests [22]. Computational models could integrate these different variables to quantify updating efficiency during course inspection, simulate terrain‐specific adaptations under fatigue, or predict tactical adjustments to variable snow conditions. Many aspects of athletic performance in alpine skiing are assessed subjectively through the coaches' eye, which may not be sensitive enough to detect small but highly significant details or mistakes [48]. Computational models provide objective, quantifiable metrics (e.g., learning rates, prediction error sensitivity, decision noise, motor variability) that can be tracked over time and used to benchmark performance, monitor recovery, or identify early signs of overtraining or injury risk.

## 7 Training and Applied Implications

Building on the Computational Sports Medicine framework, applied strategies can be designed to train a cognitive skill like updating and incorporate this with all other data. This refines individualized models, providing coaches with new tools to optimize performance and reduce injury risk. Importantly, a brain‐inclusive approach with computational sports medicine extends beyond the world's top‐ranked athletes. It offers significant opportunities for athletes positioned, for instance, in the top 50, providing them with personalized insights and targeted interventions to bridge the gap and advance into the top 20 or top 10. By acknowledging the neural underpinnings of performance, this framework is directed to a broad range of athletes to unlock their full potential.

From a coach's perspective, this means that cognitive abilities are not fixed; they are trainable, just like physical attributes. Targeted, sport‐specific drills can enhance updating by requiring athletes to adapt rapidly under uncertainty. This would help the athlete to become faster at decision‐making under pressure, reduce errors, and better adapt to unexpected conditions. Examples include simulated course inspections with time pressure, unpredictable on‐snow elements, or VR‐based decision‐making tasks [49]. Such drills can be embedded into warm‐ups or cool‐downs to emphasize deliberate practice of cognitive aspects alongside physical skills, fostering adaptability in real racing contexts.

The framework's emphasis on individualized models extends to addressing factors such as sex differences. While sex‐specific physiological adaptations are well documented in elite alpine skiers, such as lower absolute quadriceps and hamstring strength and smaller trunk muscle cross‐sectional areas in females, which may influence trunk stability and postural control [50]. Injury epidemiology also shows higher ACL and overuse injury rates among female skiers, particularly under cumulative load and fatigue conditions [2, 51, 52, 53, 54]. Governing bodies such as FIS (Fédération Internationale de Ski) address this through course design, with sex‐specific regulations on vertical drop in World Cup and championship events (FIS, 2024/2025) [55]. However, individual variability within each sex often exceeds between‐sex differences. A Computational Sports Medicine framework offers a solution by treating sex as one factor among many within individualized models, incorporating physiological (e.g., strength ratios, trunk stability, fatigue resistance) and cognitive parameters (e.g., updating efficiency, attentional flexibility). This enables personalized performance and injury‐prevention profiles rather than uniform sex‐based prescriptions. Computational Sports Medicine should be viewed as an integrative extension of existing approaches in sports medicine and performance science. It complements, rather than replaces, current biomechanical and physiological models by formalizing how cognitive and neural mechanisms interact with these established domains.

### 7.1 Integrating Motor and Cognitive Training

Motor learning research has long demonstrated that variable practice enhances adaptability and facilitates transfer of skills [56]. More recent evidence indicates that variability must be individualized rather than applied uniformly, as its effectiveness depends on learner‐specific characteristics [57]. This underscores the importance of calibrating the degree of variability to each athlete. Integrating variability in alpine skiing could include training with unpredictable gate placements, variable snow conditions (e.g., mixing icy patches with soft snow), or safely designed simulated visual impairments (e.g., partially covering the lower part of goggles to reduce surface perception). This training would force athletes to continuously update motor plans in response to changing cues. Such dual demand strengthens both motor control and cognitive updating. The coach's role will be to fine‐tune variability, challenging athletes without overwhelming them, and thereby providing structured opportunities for deliberate practice [58]. See Table 1 for examples of on‐snow training strategies to enhance cognitive and motor adaptability.

**TABLE 1:** On‐snow training strategies to enhance cognitive and motor adaptability.

Goal	Method	Description & rationale	Implementation
Enhance working memory updating & adaptability	Variable gate training	Courses with unpredictable gate placements force continuous updating of internal models and motor plans under uncertainty and fatigue	Start with short, low‐speed setups; increase complexity and speed progressively. In pre‐race periods, use small variations (e.g., shifting a single gate) to fine‐tune adaptability
Improve environmental adaptability & motor control	Controlled surface variation	Mixing ice, man‐made, and natural snow simulates course deterioration, requiring rapid motor program updates	Introduce progressively at reduced speeds. Clearly brief athletes about surface changes before runs. Ensure safety
Strengthen anticipation & processing speed	Sensory impairment drills	Restrict vision (e.g., tape on goggles) to force reliance on anticipation and internal models	Implement at lower speeds in controlled settings. Pair with inspection training and compare impaired vs. normal runs
Strengthen automaticity under cognitive load	Dual‐task skiing	Athletes perform a cognitive task (e.g., counting gates aloud) while skiing, testing focus under pressure	Start on familiar, safe terrain. Keep tasks challenging but safe; use to assess performance under distraction

### 7.2 Technology‐Enhanced Practice

Virtual reality (VR) is increasingly explored as a tool for alpine skiing training, offering controlled environments to enhance skill acquisition, feedback, and decision‐making [59, 60, 61]. Research has demonstrated the value of VR ski simulators for visualizing expert motion, manipulating temporal dynamics, and testing feedback modalities, leading to improvements in motor learning and reaction times [62, 63]. Current simulators already replicate World Cup courses [64] and allow manipulation of visual and temporal parameters. With the integration of wearable sensors, they can also provide validated kinematic and force data, adding objective metrics relevant to on‐snow performance [65, 66].

Beyond physical simulation, these VR environments also offer platforms for mental rehearsal and motor imagery training. Athletes can mentally practice complex sequences, visualize optimal lines, and anticipate environmental changes. This cognitive strategy is often suggested to enhance performance and accelerate skill acquisition. However, it is crucial to adopt a critical perspective on its application. Studies indicate that the neural representations activated during imagined actions do not always perfectly overlap with those engaged during actual physical execution [67, 68, 69]. Relying heavily on motor imagery risks creating a separate representation within the visual system, which may not directly translate to improved physical performance [70].

While still largely limited to mechanical, kinematic, and visual feedback, extending these systems to incorporate environmental unpredictability, such as fog, falling snow, or variable snow textures, would create more ecologically valid platforms for training adaptive motor and cognitive responses. Importantly, these technologies generate continuous, objective data that can be integrated into Computational Sport Medicine models, refining predictions of performance and injury risk. This would allow coaches to objectively track an athlete's updating efficiency, identify potential bottlenecks, and tailor specific training interventions. For example, if a model indicates that a skier struggles with rapid visual processing under flat light, a VR scenario could specifically be designed to target this issue. Such targeted VR training would serve as a preparatory step by enhancing the cognitive readiness for subsequent on‐snow practice in the actual challenging condition. See Table 2 for off‐snow and technology‐enhanced training examples for performance optimization.

**TABLE 2:** Off‐snow and technology‐enhanced training for performance optimization.

Goal	Method	Description & rationale	Implementation
Accelerate decision‐making & prime updating	Cognitive warm‐ups	Short, simulated inspections with unexpected changes to practice rapid mental adjustment	Integrate into warm‐ups and cool‐downs as cognitive “activation” drills to treat these components of performance as trainable as physical skills
Refine motor programs & simulate scenarios	Virtual reality (VR) training	VR allows safe manipulation of variables (visibility, snow, gate timing) to train adaptability	Use for scenario‐based training and “what‐if” simulations without physical load
Build individualized performance & injury‐risk profiles	Integrated data collection	Combine sensor, cognitive, physiological, and psychological data into personalized models	Apply within a computational sports medicine framework for tailored feedback

## 8 Future Directions

**FIGURE 2:**
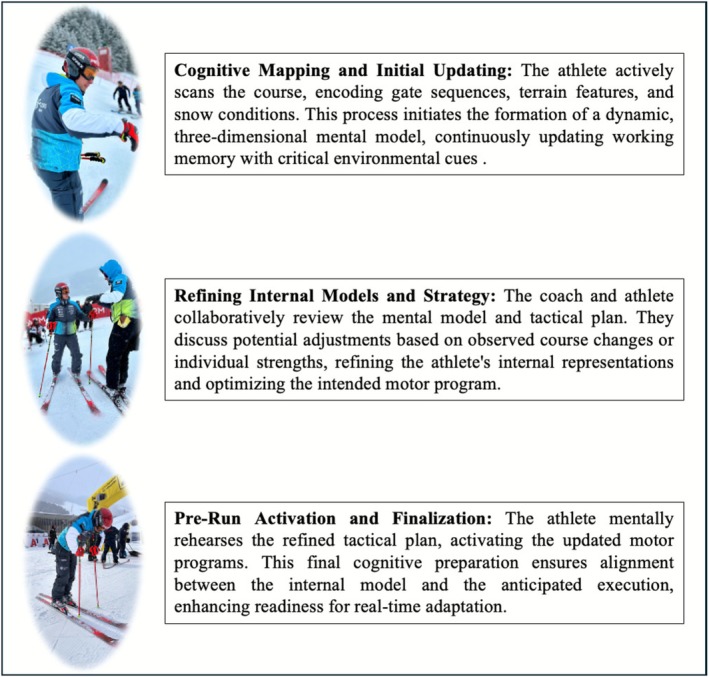
Athlete and coach collaboration when applying computational sports medicine in Alpine skiing. This figure illustrates the practical, iterative process of integrating cognitive and motor preparation in alpine skiing, emphasizing the collaboration between athlete and coach within the Computational Sports Medicine framework. It highlights how cognitive processes, particularly updating, are refined and translated into actionable motor plans.

Integrating cognitive neuroscience with Computational Sports Medicine opens clear avenues for research and practice. See Figure 2 for an illustration of the athlete and coach collaborative effort within the Computational Sports Medicine framework. The next step is to design and validate cognitive tasks that closely mirror alpine skiing demands, for example, through VR simulations featuring dynamic course variations and unpredictable sensory conditions. Such tasks would enable precise, ecological measurements of updating performance and decision making under real performance constraints. These tasks should then be used in intervention studies to test whether adaptive working memory training, VR‐based drills, or other cognitive protocols translate into measurable gains in performance and adaptability on snow.

At the computational level, Bayesian inference, reinforcement learning, and active inference models can formalize how skiers update internal models (the brain's understanding of body dynamics and environmental interactions) during both course inspection and race execution. The long‐term goal of Computational Sports Medicine is to integrate cognitive, biomechanical, and physiological states into predictive models that can forecast performance outcomes and injury risk. Longitudinal studies will be essential to capture how cognitive abilities, physical development, and performance trajectories interact across an athlete's career, ultimately translating theory into individualized training and rehabilitation strategies.

## 9 Conclusion

This review argues for a paradigm expansion in sports medicine, moving beyond the traditional “shoulders downward” perspective to fully recognize the brain's central role in athletic performance and health. We propose Computational Sports Medicine as a framework that integrates cognition, biomechanics, and physiology through rigorous computational modeling. This approach transitions sports medicine from reactive, generalized interventions to proactive, personalized strategies for performance optimization, injury prevention, and return‐to‐play decisions. By quantifying brain and cognitive processes and integrating multiscale data, this framework can generate the predictive insights necessary for such personalization. Although alpine skiing serves as our main case, the Computational Sports Medicine framework can be applied to other high‐speed, feedback‐rich sports such as motocross, sailing, or tennis, where updating and prediction‐error minimization are also critical. Situating the brain as the central organ of athletic performance, adaptation, and rehabilitation is not only natural but necessary for the future of sports medicine.

## Funding

The authors have nothing to report.

## Conflicts of Interest

The authors declare no conflicts of interest.

## Data Availability

Data sharing not applicable to this article as no datasets were generated or analyzed during the current study.
